# The General Practitioner's Indictment of the Hospitals

**Published:** 1888-07-07

**Authors:** 


### The Hospital

A Weekly Institutional Journal of

**Science, Medicine, Nursing, and Philanthropy.**

*For Hospitals, Asylums, Medical Practitioners, Students, Nurses, Families, Parishes, Congregations, and the Charitable Public.*

Vol. IV. No. 93.] [July 7, 1888.

### The General Practitioner's Indictment of the Hospitals.

The general practitioner of medicine is in a condition of acute discontent. He is ground between the upper and nether millstones of keen rivalry within his own ranks, and still more injurious competition on the part of hospitals of every kind and degree. He is, as a rule, a patient drudge, plodding on at his daily and exacting toil with no more serious revolt in his soul than can be blown away by a good, all-round grumble. But the longsuffering man sometimes proves both fierce and untameable when fairly roused; and there are not wanting a good many indications that the patience of the general practitioner is coming to an end. The world is undoubtedly his oyster, as it is other men's; but he often finds it not only very difficult to open at the outset, but a very poor oyster indeed when opened. The labour of its deglutition and digestion are both of the toughest, and the amount of nutrition yielded to himself and his family is often the very smallest compatible with survival.

Given an oyster which, in spite of all efforts to open it, will persist in remaining "obstinately shut," and of such a nature that when opened it is neither juicy nor sweet, what wonder is it that the opener scowls and looks unpleasant when he sees somebody else waiting until it is opened to take the lion's share? That is what the general practitioner conceives to be the situation as between himself and the hospital system of this country. Of all his rivals he regards hospitals as the keenest; of all his competitors they compete under most advantages, and are therefore most likely to be first at the winning-post. Here is what Mr. David Walsh, a *bonâ-fide* general practitioner, says on the subject: "The hospital authorities, hitherto content with the sprats, are now casting about for the salmon, careless what rights they may be invading. . . . The general practitioner, with his disunited forces, is a mark for plunder on all hands, and it is in vain he turns for help to the medical corporations, the hospitals, or the medical press. . . . A short time back the statement was circulated that the life of a doctor fresh from graduation was not considered a good policy (for insurance) for the first four years. In other words, it takes five years to kill his ambition, to blunt his susceptibilities, and to ensure his making a living. . . . This state of affairs is in no small measure traceable to the competition of the hospitals, backed up as they are by a charity that is magnificent indeed, but none the less injurious, through a want of reasonable guidance. . . . It cannot be too strongly insisted upon that, by *charging* well-to-do hospital patients, so far from the wrong being righted, an additional injury is thrown on a learned profession, whose average income is hardly above that of a middle-class bank clerk. The general practitioner, thus ignored and fleeced by the hospitals, not unnaturally regards those institutions with distrust, suspicion, and indifference."

Such is Mr. Walsh's bold and resolute challenge to the hospitals; such the indictment they are called upon to meet and to disarm. No man who has known general practice by experience, as the writer has, will fail to sympathise entirely with Mr. Walsh in his just complaints. It is undoubtedly true that hospitals are the most injurious, because by far the most successful, of all the natural enemies of the rank and file of the medical profession. They offer to the bulk of the population, practically without check of any kind, medical resources and skill almost always equal and often superior to that of the general practitioner, and, in addition, food, lodging, and nursing incomparably better than the patient has at home. Is it to be wondered at that under these circumstances the hospital is first, and the private practitioner entirely out of the running? The artisan or the small tradesman, whose necessities impel him to seek the speediest means of cure, would be much more than an average man if his high sense of honour prevented him from availing himself of the freely offered gifts of the hospital; whilst from a utilitarian point of view he would be a fool if he did not choose that method of medical treatment which will most speedily restore him to his ordinary avocations. There is the crux of the problem. The hospitals offer, almost always as good, in serious cases often better treatment than the family practitioner. They offer this for nothing; and they add a huge bribe in the shape of perfect lodgings, food, and nursing. The patient takes the offer of the hospital, and will continue to take it to the end of time, unless something be done to prevent him. All things considered, he will do not only what is natural, but also what, from his point of view, is right in so deciding.

Everybody is agreed that the general practitioner is competed with, to his injury, by the powerful and charitably-supported hospitals; everybody admits that many people could pay a large proportion of the value of what they receive in hospitals for nothing, whilst a still larger number could pay a small proportion. What, then, is the remedy which suggests itself to the practical man? It is of this kind: No person shall be treated gratuitously at hospitals until he has proved his poverty. He who, not being absolutely poor, may still rightly claim hospital treatment on other grounds must pay for it in proportion to his means. So say those who have a not unreasonable faith in the general principles of political economy. "No," say the general practitioners, "that would only be making matters worse." Who is right, the political economists or the doctors? On the first blush it would seem that the doctors must be wrong. If the laws which regulate medical practice in all its departments be the same as those which regulate all the other inter-relations of human activity, the economists must be right. If the economists are wrong, then we have brought into play in one sphere of life a principle the exact opposite of that which prevails in every other sphere. In every other department, if a man have an unsatisfied need he will satisfy it as fully as he can, and the less the cost of its satisfaction the more fully will he satisfy it; the greater the cost the less fully will he satisfy it. No one will deny the soundness of this proposition, or its general applicability. "Yes," say the doctors, "that is generally, indeed almost universally true; but in matters medical the exact opposite will obtain. If a man needs medical treatment, he will seek it readily and constantly if he has to pay the full or the part value of it; but he will seek it much less readily if he can get it for nothing at all."

Now that position seems to us to labour under the disadvantages of being not only contrary to the principles of economic science, which is generalised experience, but contrary also to common sense. If its soundness can be proved by indisputable facts, we shall admit it, but not otherwise. We think, moreover, that the majority of mankind will be entirely on the side of the economists, at any rate until their conversion is effected by the most convincing arguments. The pay system judiciously administered would, we hold, be advantageous to patients, more advantageous to hospitals, and most of all advantageous to the general practitioners of medicine. It would diminish the injurious competition of hospitals in exact proportion to the thoroughness of its application. Such, at any rate, is the judgment of plain sense, and such, too, is the experience of foreign countries, and of those hospitals which have tried the pay system in this country. Taking the present income of the medical profession, including the hospitals, as a whole, at twenty millions a-year, a thoroughly-applied pay system would probably raise it to at least twenty-one millions. The question is, will the whole profession, and each of its members, be more prosperous by the distribution among them of twenty millions or of twenty-one millions?

